# Deepening the LUMO: Brominated Naphthalene Diimide Electron Transport Layers for Low-Hysteresis Perovskite Solar Cells

**DOI:** 10.1021/acs.chemmater.5c02132

**Published:** 2025-11-19

**Authors:** Sanggyun Kim, Justine S. Wagner, Sina Sabury, Spencer J. Gilman, Jack Lawton, D. Eric Shen, Anna M. Österholm, Carlo A. R. Perini, John R. Reynolds, Juan-Pablo Correa-Baena

**Affiliations:** † School of Materials Science and Engineering, 1372 Georgia Institute of Technology, Atlanta, Georgia 30332, United States; ‡ School of Chemistry and Biochemistry, Center for Organic Photonics and Electronics, 1372 Georgia Institute of Technology, Atlanta, Georgia 30332, United States

## Abstract

Precise energy level alignment at the interfaces between the charge transport layers, active layer, and electrodes plays a key role in maximizing photovoltaic performance and operational stability in perovskite solar cells (PSCs). Organic electron transport layers (ETLs) have received little attention compared to their inorganic counterparts but offer the unique advantage of facile functionalization for fine-tuning of electronic properties. Here, we report the design, synthesis, and characterization of two benzyl-phosphonic acid (BnPA)-functionalized naphthalene diimide (NDI) derivatives, NDI-(BnPA)_2_ and Br_2_-NDI-(BnPA)_2_ as organic ETLs for PSCs in an n-i-p device configuration. Bromination of the NDI core at the 4,9-positions deepens the lowest unoccupied molecular orbital (LUMO) in Br_2_-NDI-(BnPA)_2_ by 0.29 eV, enabling improved energy alignment with the conduction band minimum of the perovskite. Devices incorporating Br_2_-NDI-(BnPA)_2_ demonstrated enhanced short-circuit current density (*J*_SC_), reduced hysteresis, and a maximum power conversion efficiency of 13.67%, compared to 13.20% for the unsubstituted NDI-(BnPA)_2_ analog. The deeper LUMO of Br_2_-NDI-(BnPA)_2_ is hypothesized to facilitate more efficient electron extraction, suppress interfacial charge accumulation, and reduce field-driven ion migration, collectively contributing to the observed reduction in hysteresis. These results highlight the effectiveness of combining molecular-level LUMO tuning with robust interfacial anchoring to advance the performance and durability of organic ETLs in PSCs.

## Graphical abstract

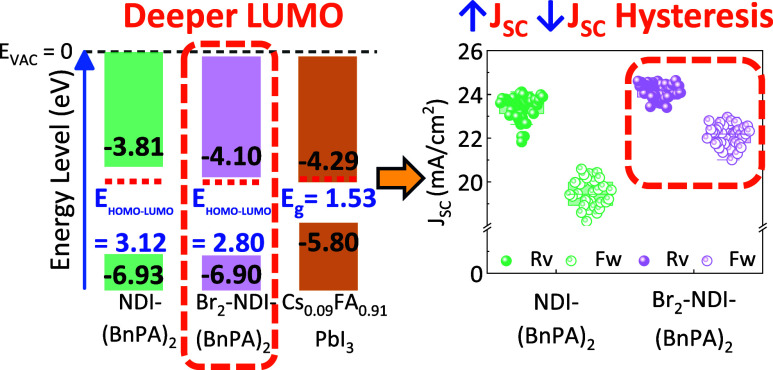

## Introduction

Perovskite solar cells (PSCs) have gained significant attention due to their high power conversion efficiencies (PCEs) and the potential for low-cost, solution-based fabrication.^1^ Continued improvements in both performance and stability are constrained by interfacial losses, primarily caused by imperfect energy level alignment and interfacial recombination.^2,3^ Aligning the energy levels of the charge transport layers (CTLs), perovskite absorber, and metal electrodes is essential to ensure efficient carrier extraction and minimal recombination losses. Energetic misalignment at these interfaces can introduce potential barriers, impede charge transfer, and elevate nonradiative recombination, leading to a reduction in open-circuit voltage (*V*_OC_), short-circuit current density (*J*_SC_), fill factor (FF), and overall device efficiency.^4,5^ Beyond steady-state transport limitations, poor energy alignment also promotes local charge accumulation, which distorts internal electric fields and leads to the migration of mobile ionic species, predominantly halides, in the perovskite lattice.^6,7^ Although ionic motion is distinct from electronic conduction, it is governed by internal electric fields that intensify under inefficient charge extraction. Accumulated interfacial charges amplify these fields, accelerating the migration of halides and contributing to dynamic instabilities such as *J–V* hysteresis and long-term performance degradation in PSCs.^6,8–11^ Hysteresis in PSCs is a significant issue, as it leads to discrepancies in *J–V* measurements, which can result in under- or overestimations of PCE, challenges in determining the maximum power point voltage, and complications in tracking device performance over time. Thus, energy level alignment is not only necessary for charge extraction and suppressing recombination but also for stabilizing the internal electric field environment to ensure reliable PSC operation.

Among CTLs, the electron transport layer (ETL) plays a central role in selectively extracting electrons while blocking holes, thereby minimizing recombination losses at the perovskite interface. Inorganic ETLs such as TiO_2_ and SnO_2_ have been widely adopted due to their favorable conduction band alignment and intrinsic material stability.^12,13^ However, their performance is constrained by structural and interfacial limitations. TiO_2_ typically requires high-temperature processing (>450 °C) to achieve adequate crystallinity and electronic conductivity, which limits its compatibility with flexible substrates and low-cost manufacturing.^14^ SnO_2_ offers lower-temperature (<200 °C) processability but often suffers from oxygen vacancies and under-coordinated tin sites, which can introduce surface trap states, facilitate recombination, and ultimately reduce *V*_OC_.^15,16^ These challenges have spurred growing interest in organic ETLs, which offer molecular-level tunability, low-temperature processability, and compatibility with scalable, solution-based deposition methods. Despite these advantages, organic ETLs present their own challenges. Poor interfacial adhesion to transparent conductive oxides (TCOs) such as fluorine-doped tin oxide (FTO) or indium tin oxide (ITO) can result in partial desorption or dissolution during perovskite deposition or prolonged device operation, undermining interfacial stability.^17,18^

NDI-based frameworks have been widely recognized for their electron-deficient character, ambient stability, and propensity for π–π stacking, making them attractive candidates for ETL design.^19–22^ Additionally, NDIs can be functionalized either at the imide nitrogen or on the conjugated core, allowing several strategies for synthetically tuning their properties. One prior study reported the use of phosphonic acid (PA) anchoring groups onto NDI molecules, highlighting their beneficial role in enhancing interfacial adhesion with TCOs and ensuring consistent charge transfer and device stability.^23^ However, this study did not explicitly examine how anchoring groups on the organic ETL affect overall PSC device stability. Motivated by this knowledge gap, we have explored PA-functionalized NDI-based small molecules as ETLs to probe the role of anchoring groups in ensuring long-term operational stability.^24^ Although the methylene-linked PA groups in those molecules introduce conformational flexibility that could, in principle, enable dual binding, our work has shown that phosphonic acids reliably improve adhesion and stability even if precise orientation cannot be resolved.^24^ Specifically, we investigated ((1,3,6,8-tetraoxo-1,3,6,8-tetrahydrobenzo­[lmn]­[3,8]­phenanthroline-2,7-diyl)­bis­(4,1-phenylene))­bis­(phosphonic acid) (NDI-(PhPA)_2_) and 2,7-bis­(4-bromophenyl)­benzo­[lmn]­[3,8]­phenanthroline-1,3,6,8­(2H,7H)-tetraone (NDI-(PhBr)_2_) as ETLs within an n-i-p device configuration. The bromophenyl functional groups in NDI-(PhBr)_2_ were designed as a nonanchored control, relying exclusively on weak physisorption to the TCO surface, to demonstrate the impact of anchoring groups on ETL interfacial stability. Consistent with a prior report, NDI-(PhPA)_2_ demonstrated strong interfacial bonding with TCOs and robust chemical resistance during perovskite processing, but this improved adhesion does not directly translate into higher efficiencies. Instead, reduced *V*_OC_ and pronounced *J–V* hysteresis are attributed to unfavorable energy level alignment, where the LUMO of NDI-(PhPA)_2_ lies above the perovskite CBM, creating an extraction barrier and promoting interfacial charge accumulation.

In this study, we leverage the synthetic tunability of NDIs to design two new ETLs, varying the energy of the LUMO levels and evaluating how energy level alignment with the perovskite layer impacts device performance. These molecules, ((1,3,6,8-tetraoxo-1,3,6,8-tetrahydrobenzo­[lmn]­[3,8]­phenanthroline-2,7-diyl)­bis­(4,1-phenylene))­bis­(methylene))­bis­(phosphonic acid) (NDI-(BnPA)_2_) and its dibrominated analogue (((4,9-dibromo-1,3,6,8-tetraoxo-1,3,6,8-tetrahydrobenzo­[lmn]­[3,8]­phenanthroline-2,7-diyl)­bis­(4,1-phenylene))­bis­(methylene))­bis­(phosphonic acid) (Br_2_-NDI-(BnPA)_2_), retain the anchoring functionality of PhPA while introducing a methylene spacer for improved solubility and processability. The two BnPA groups serve complementary roles: one provides strong chemical adhesion to TCOs, while the second enhances surface wettability to promote uniform perovskite film coverage. π-Conjugated core modification with electron-withdrawing substituents, such as halogens, nitriles, or acyl groups, has previously been employed to lower the LUMO energy level and improve interfacial alignment.^19^ We have introduced bromine atoms on the NDI core of Br_2_-NDI-(BnPA)_2_, where their electronegativity lowers the NDI’s LUMO level, in order to improve alignment with the perovskite conduction band minimum (CBM) and facilitate more efficient electron extraction. Ultraviolet photoelectron spectroscopy (UPS) confirmed a LUMO downshift in the brominated derivative, while UV–vis absorption measurements indicated a reduction in the highest occupied molecular orbital (HOMO)-LUMO gap. When integrated into planar n-i-p PSCs, devices employing Br_2_-NDI-(BnPA)_2_ as the ETL achieved a maximum PCE of 13.67% (maximum stabilized PCE of 12.55%), outperforming the 13.20% (maximum stabilized PCE of 10.93%) obtained with NDI-(BnPA)_2_. The improved performance is attributed to more favorable energy alignment at the perovskite interface and enhanced electron extraction enabled by a deeper LUMO level. More importantly, Br_2_-NDI-(BnPA)_2_-based devices also suppressed *J*_SC_ hysteresis, further supporting the hypothesis that deeper LUMO levels reduce interfacial charge accumulation and field-driven ion migration. Both ETLs maintained stable output under continuous 1 sun illumination at 25 °C for over 200 h, demonstrating the synergistic role of anchoring group integration and energetic tuning in enabling high-performance, stable organic ETLs for PSCs.

## Results and Discussion

### Synthesis and Thermal Characterization of NDI-(BnPA)_2_ and Br_2_NDI-(BnPA)_2_

Figure 1a illustrates the molecular structures of NDI-(BnPA)_2_ and Br_2_-NDI-(BnPA)_2_. Synthetic procedures are outlined in Figures S1 and S2, and their structures were verified by nuclear magnetic resonance spectroscopy (NMR) (Figures S3–S12). The thermal stability of both molecules was evaluated by thermogravimetric analysis (TGA) (Figure 1b), which revealed that both molecules exhibit less than 0.5% mass loss up to 200 °C (inset). This indicates that the molecules maintain their structural integrity well above the typical perovskite solution processing conditions and annealing temperature of 150 °C, confirming their suitability for PSC fabrication and operation. Above 200 °C, distinct mass loss events are observed, which are likely associated with evaporation or thermal degradation processes. Furthermore, complementary differential scanning calorimetry (DSC) was performed on the precursor powders of both molecules (Figure S13). The second DSC heating cycle, which excludes effects from residual solvents or relaxation captured in the first cycle, revealed no discernible phase transitions, such as melting or crystallization, up to 250 °C (Figure S13). The absence of phase transitions in the second cycle is encouraging, as phase changes could lead to morphological instabilities, including crack formation or interfacial desorption during device operation.^25^ These results demonstrate that both NDI derivatives possess the thermal robustness necessary for their stable integration into PSCs.

**Figure 1:**
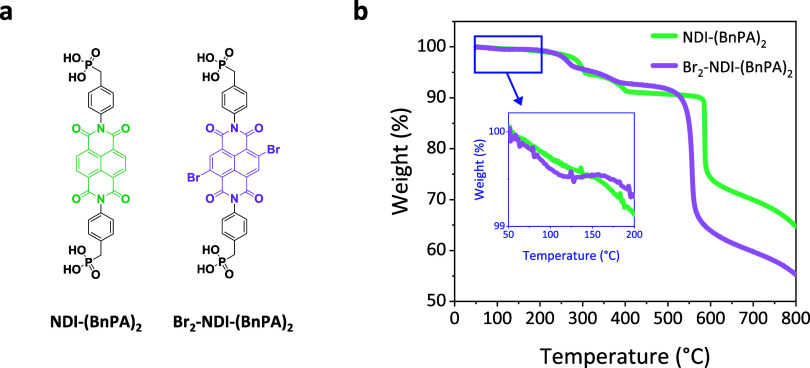
Molecular structures and TGA thermograms of NDI-based ETLs. (a) Molecular structures and (b) thermograms of NDI-(BnPA)_2_ and Br_2_-NDI-(BnPA)_2_ molecules. The inset shows the 50–200 °C range relevant to PSC processing conditions, within which both ETLs demonstrate excellent thermal stability, exhibiting less than 0.5% mass loss likely due to residual solvent.

### Deposition and Solvent Resistance of NDI-(BnPA)_2_ and Br_2_-NDI-(BnPA)_2_ Thin Films

Thin films of NDI-(BnPA)_2_ and Br_2_-NDI-(BnPA)_2_ were deposited onto various substrates, including glass, FTO, and ITO, using a chemical bath deposition (CBD) process. Each molecule was dissolved in dimethyl sulfoxide (DMSO) at a concentration of 0.5 mg mL^–1^. The precleaned substrates were then immersed into the heated CBD solution at 100 °C for 72 h to promote uniform chemisorption onto the substrate surfaces. Following deposition, the substrates were dipped in ethanol to remove unbound molecules and then thermally annealed at 120 °C for 10 min to eliminate residual solvent. A detailed description of the CBD method is available in our previous report.^24^

To evaluate molecular deposition, X-ray photoelectron spectroscopy (XPS) was performed on bare FTO, and on NDI-(BnPA)_2_-coated and Br_2_-NDI-(BnPA)_2_-coated FTO substrates (Figure 2). The P 2p signal (Figure 2a) of bare FTO displayed a peak at 139 eV. Upon molecular deposition, two distinct changes were observed: (i) attenuation of the FTO-derived 139 eV signal and (ii) appearance of a new peak centered at 133.9 eV, characteristic of phosphorus from the phosphonic acid anchoring groups of the NDI-based molecules. The emergence of the phosphorus signal confirms the deposition of the ETL molecules on FTO. Further molecular differentiation was achieved through Br 3d analysis (Figure 2b). A Br 3d doublet between 71 and 72 eV was detected exclusively for the Br_2_-NDI-(BnPA)_2_ thin film, consistent with the presence of bromine substituents incorporated within the molecular structure. No corresponding Br 3d signal was observed for the bare FTO or NDI-(BnPA)_2_ thin film, validating the molecular distinction of the brominated molecule. Finally, the N 1s spectra (Figure 2c) exhibited strong peaks centered around 401 eV for both molecular films, corresponding to the nitrogen of the NDI core. In contrast, bare FTO showed only a weak, broad feature near 399 eV. Collectively, these results demonstrate the successful CBD of the NDI-based ETLs on FTOs.

**Figure 2:**
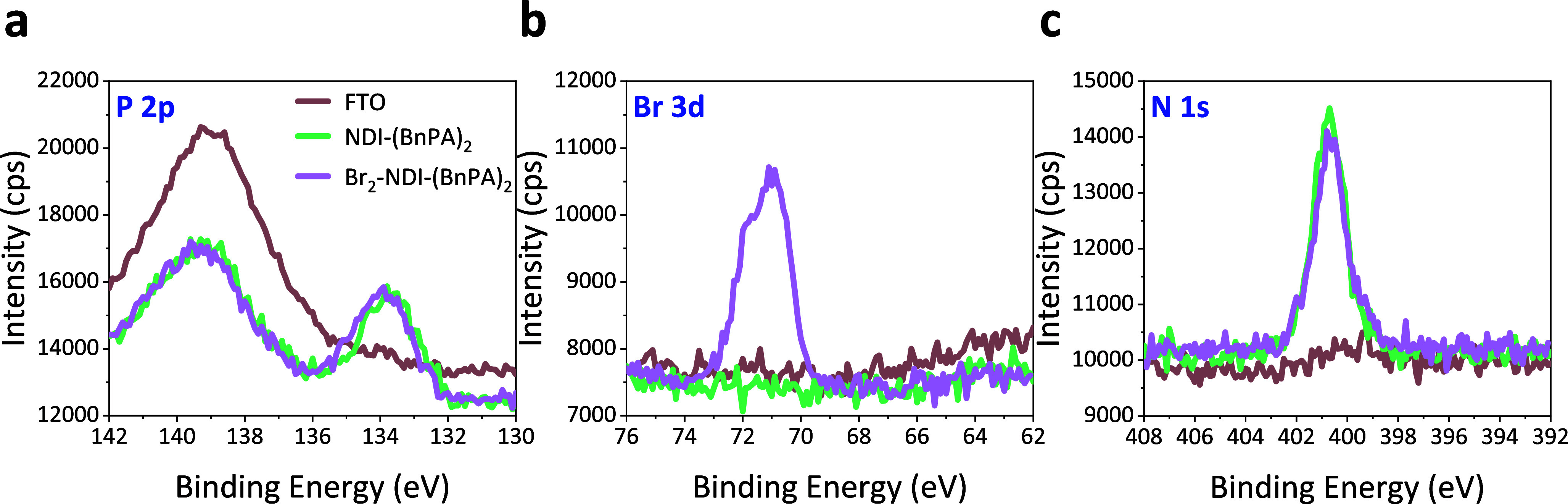
XPS analysis of NDI-based ETL deposition on FTO. XPS spectra of (a) P 2p, (b) Br 3d, and (c) N 1s for NDI-(BnPA)_2_ and Br_2_-NDI-(BnPA)_2_ films deposited on FTO substrates.

Given that DMSO is a common solvent both in the CBD process and in perovskite precursor solutions, evaluating the robustness of the molecular thin films under solvent exposure is critical to assess their stability in PSC fabrication. To this end, a solvent washing test was conducted, wherein pristine NDI-(BnPA)_2_ and Br_2_-NDI-(BnPA)_2_ thin films on FTO were exposed to solvents by sequentially spin-coating a 90 μL solution of dimethylformamide (DMF):DMSO (2:1 v/v) followed by 250 μL of chlorobenzene (CB). This wash test was designed to replicate the solvent environment experienced during the preparation of perovskite films via spin-coating. XPS characterization following the solvent washing revealed no change in the P 2p signal for either the NDI-(BnPA)_2_ or Br_2_-NDI-(BnPA)_2_ thin films, indicating that the phosphonic acid anchoring groups remained strongly bound to the FTO surface (Figure 3a). Similarly, no shifts or changes in intensity were observed for the N 1s peaks of either film and the Br 3d peaks in Br_2_-NDI-(BnPA)_2_ spectra remain unaltered (Figures 3b,c and S14), further supporting the stability of the molecular films. These findings demonstrate that NDI-(BnPA)_2_ and Br_2_-NDI-(BnPA)_2_ form chemically robust and mechanically resilient interlayers capable of withstanding solvent processing, an essential requirement for ensuring stable interfaces in PSCs.

**Figure 3:**
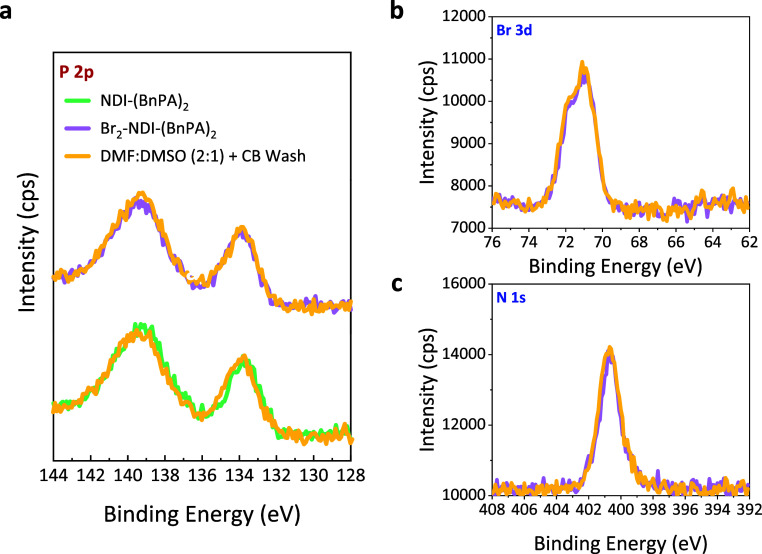
XPS analysis of NDI-based ETL retention on FTO after solvent washing. XPS spectra of (a) the P 2p region for NDI-(BnPA)_2_ and Br_2_-NDI-(BnPA)_2_, (b) the Br 3d region, and (c) the N 1s region for Br_2_-NDI-(BnPA)_2_ films on FTO, before and after washing with DMF:DMSO (2:1) and CB solvents to simulate perovskite spin-coating conditions and assess molecular retention.

### Impact of Dibromo Substitution on the Optoelectronic Properties of NDI-Based ETLs

The optical properties of NDI-(BnPA)_2_ and Br_2_-NDI-(BnPA)_2_ were assessed via UV–vis spectroscopy in both solution (Figure S15) and thin films (Figure 4), using bare glass as a reference to isolate molecular absorption. NDI-(BnPA)_2_ thin films exhibited distinct absorption peaks at 362 and 381 nm, whereas Br_2_-NDI-(BnPA)_2_ thin films showed red-shifted absorption features at 394 and 416 nm (Figure S15b). This redshift reflects a reduction in the optical HOMO–LUMO gap due to bromination of the NDI core, as confirmed by Tauc plot analysis. In solution (Figure S15b), NDI-(BnPA)_2_ exhibited sharp, well-defined absorption peaks at 362 and 381 nm, consistent with its thin-film spectrum. This suggests minimal aggregation in solution and preservation of its molecular electronic structure during film formation. In contrast, Br_2_-NDI-(BnPA)_2_ displayed broadened and convoluted absorption features centered around 363 and 400 nm in solution, which did not correspond directly to the distinct absorption peaks observed in its thin-film state. The optical HOMO–LUMO gaps were determined to be 3.12 eV for NDI-(BnPA)_2_ and 2.80 eV for Br_2_-NDI-(BnPA)_2_, as shown in Figure 4a,b insets.

**Figure 4:**
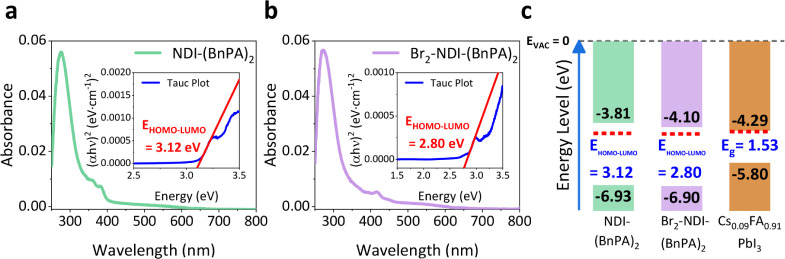
Optoelectronic characterization and energy level alignment of NDI-based ETLs. UV–vis absorption spectra of (a) NDI-(BnPA)_2_ and (b) Br_2_-NDI-(BnPA)_2_ thin films deposited on glass substrates, along with Tauc plot extrapolations (insets) to estimate their optical LUMO–HOMO energy gaps. (c) Energy level diagram of NDI-based ETLs and halide perovskite. The red dashed lines indicate the Fermi levels of each material.

Figure 4c presents the energy level alignment diagram for the Cs_0.09_FA_0.91_PbI_3_ perovskite, NDI-(BnPA)_2_, and Br_2_-NDI-(BnPA)_2_ thin films. UPS was performed on each thin film deposited on an ITO substrate to extract the work functions and HOMO/valence band maximum levels (detailed energy positions are shown in Figure S17 and Table S1). By combining UPS results with optical HOMO–LUMO gap values obtained from UV–vis measurements, a complete energy level diagram was constructed. The optical energy bandgap of Cs_0.09_FA_0.91_PbI_3_ was determined to be 1.53 eV (Figure S16). Bromination lowered the LUMO level from −3.81 eV for NDI-(BnPA)_2_ to −4.10 eV for Br_2_-NDI-(BnPA)_2_, while the HOMO levels remained largely unchanged. The LUMO offset between NDI-(BnPA)_2_ and the perovskite CBM is approximately 0.48 eV, whereas Br_2_-NDI-(BnPA)_2_ achieved a smaller offset of 0.19 eV. Although both LUMO levels lie above the perovskite CBM, the reduced offset in Br_2_-NDI-(BnPA)_2_ is expected to facilitate more efficient electron extraction and lower interfacial electron accumulation, which can help suppress hysteresis under operation conditions at room temperature. It is worth noting that while UPS measurements provide valuable insights into relative energy levels, the absolute values may vary slightly depending on the experimental conditions. However, the relative energy differences between the materials measured under identical conditions remain reliable for comparison. Additionally, the HOMOs of both molecules are comparable with previously reported values for NDI derivatives, estimated at approximately −6.9 eV from cyclic voltammetry, aligning closely with our UPS measurements.^26^

### Photovoltaic Performance and Stability of NDI-Based ETL Devices

To evaluate the impact of varying the LUMO in the NDI-based ETLs, PSCs were fabricated in a conventional n-i-p planar configuration comprising FTO/ETL/Cs_0.09_FA_0.91_PbI_3_/PEAI/doped Spiro-OMeTAD/Au (Figure S18). Devices fabricated without an ETL (w/o ETL), with NDI-(BnPA)_2_, and with Br_2_-NDI-(BnPA)_2_ were compared. Figure 5a shows the *J*_SC_ under reverse and forward bias sweeps for the different ETLs. While the *V*_OC_ and FF remained similar between devices utilizing NDI-(BnPA)_2_ and Br_2_-NDI-(BnPA)_2_, a slight increase in *J*_SC_ and a pronounced reduction in *J*_SC_ hysteresis were observed for Br_2_-NDI-(BnPA)_2_ (Figure S19a,c). The improvement is attributed to the deeper LUMO of Br_2_-NDI-(BnPA)_2_ (−4.10 eV), which reduces the energy offset with the Cs_0.09_FA_0.91_PbI_3_ CBM (−4.29 eV), enhancing electron extraction. Although dibromo substitution improves LUMO alignment and facilitates more efficient electron extraction, the similar Fermi levels of NDI-(BnPA)_2_ and Br_2_-NDI-(BnPA)_2_ maintain comparable built-in potentials, resulting in unchanged *V*_OC_ and FF across the two devices. We note that *V*_OC_ in PSC is primarily determined by quasi-Fermi level splitting inside the perovskite, not solely by the ETL-perovskite energy alignment, which might explain the same *V*_OC_ observed for the two interlayers.^27,28^ Devices without an ETL exhibited the highest *J*_SC_ hysteresis, *V*_OC_, and FF, consistent with increased surface recombination and inefficient charge extraction due to direct perovskite/FTO contact. Stabilized PCEs (Figure 5b) followed the expected trend: Br_2_-NDI-(BnPA)_2_ (12.55%; median 10.55%) > NDI-(BnPA)_2_ (10.93%; median 9.49%) > w/o ETL (8.04%; median 6.04%), further supporting the role that an optimized LUMO alignment has in improving performances via enhanced carrier extraction. Detailed photovoltaic parameters are provided in Figure S19 and Table S2. Further insight into the interfacial charge dynamics is provided by the hysteresis index (HI) extracted from the reverse and forward *J–V* scans (Figure 5c). The HI, calculated as the ratio of PCE obtained from the forward and reverse scans, can be used to quantify the discrepancy in the *J–V* scans between different devices. Devices incorporating Br_2_-NDI-(BnPA)_2_ exhibited a minimum HI of 0.26, which is significantly lower than the minimum HI value of 0.33 observed for devices utilizing NDI-(BnPA)_2_, highlighting improved interfacial charge extraction. In contrast, devices without an ETL showed the highest HI minimum value of 0.66, underscoring the importance of proper interfacial energy alignment and carrier selectivity for reducing hysteresis. Detailed HI values are summarized in Table S3.

**Figure 5:**
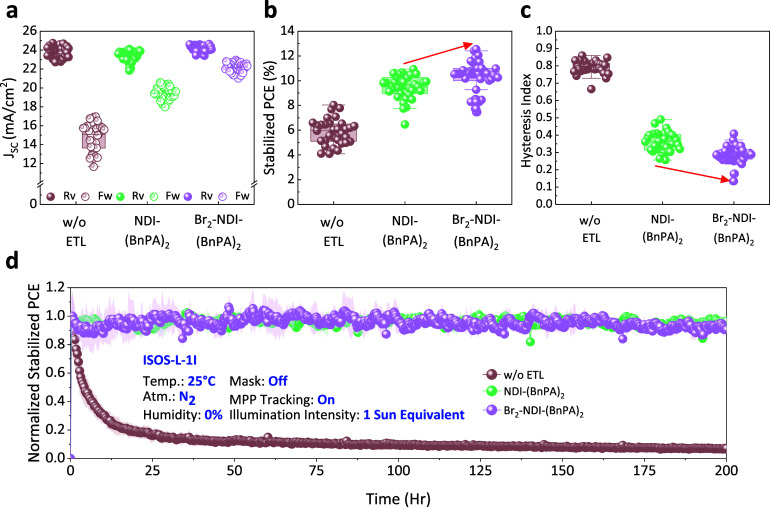
Photovoltaic performance and operational stability of PSCs with NDI-based ETLs. (a) *J*_SC_ under both reverse and forward scan directions, (b) stabilized PCE, and (c) HI of PSCs incorporating ETL-free (w/o ETL), NDI-(BnPA)_2_, and Br_2_-NDI-(BnPA)_2_. HI quantifies the degree of *J–V* scan direction dependence of the PCE, calculated as the relative difference between reverse (positive to negative bias) and forward (negative to positive bias) scan PCEs. (d) Long-term stability of devices under continuous AM 1.5G illumination and MPPT at 25 °C in a nitrogen atmosphere, following the ISOS-L-1I protocol.

Finally, long-term operational stability was assessed following the International Summit on Organic PV Stability (ISOS)-L-1I protocol.^29^ Devices were subjected to continuous maximum power point tracking (MPPT) under 1 sun equivalent illumination at 25 °C in an N_2_ atmosphere (Figure 5d), with *J–V* scans recorded every 12 h (Figure S20). Over 200 h of continuous operation, the normalized stabilized PCEs of NDI-(BnPA)_2_ and Br_2_-NDI-(BnPA)_2_ integrated devices remained largely unchanged, demonstrating excellent operational durability. In contrast, devices fabricated without an ETL exhibited rapid performance degradation, reaffirming the importance of the ETL layer in protecting the perovskite interface and maintaining device integrity during prolonged operation.

## Conclusion

In this work, we demonstrate the rational design and implementation of two NDI-based ETLs, NDI-(BnPA)_2_ and Br_2_-NDI-(BnPA)_2_, for use in PSCs. Bromine addition to the NDI core of the molecule effectively lowers the LUMO of Br_2_-NDI-(BnPA)_2_ while preserving the film integrity, resulting in improved energy alignment with the Cs_0.09_FA_0.91_PbI_3_ perovskite conduction band maximum. Devices incorporating Br_2_-NDI-(BnPA)_2_ achieved a maximum PCE of 13.67% (maximum stabilized PCE of 12.55%) compared to 13.20% (maximum stabilized PCE of 10.93%) of solar cells utilizing NDI-(BnPA)_2_. The PCE improvement is driven by enhanced *J*_SC_ and reduced *J*_SC_ hysteresis, while maintaining similar *V*_OC_ and FF owing to comparable Fermi levels. Notably, both ETLs maintained operational stability under continuous illumination at 25 °C for 200 h, confirming the role of anchoring PA groups in preserving interfacial robustness.

Despite these advances, UPS and UV–vis analyses reveal that the LUMO of both ETLs remains slightly higher than the CBM of Cs_0.09_FA_0.91_PbI_3_, suggesting a still suboptimal energy alignment. To address this, further electronic tuning, such as extended halogenation or integration of alternative electron-withdrawing substituents, may be required to achieve ideal alignment. These findings underscore the value of simultaneously tuning ETL energetics and interface bonding mechanisms to optimize device performance and reliability, offering encouraging directions for the continued development of high-efficiency, solution-processable organic ETLs for next-generation PSCs and beyond.

## Experimental Methods

### Molecule Synthesis

The synthesis of the molecules was carried out according to the method described in the Supporting Information.

### PSC Fabrication

Patterned FTO glass substrates (7 Ω/sq) were cleaned sequentially via ultrasonication in 2% Mucasol (Schülke) solution, deionized water, acetone (≥99.5%, Sigma-Aldrich), and isopropyl alcohol (IPA, 99.9%, Fisher Chemical), each for 15 min. After drying with a N_2_ gun, substrates were treated with UV-ozone for 15 min. For devices incorporating TiO_2_-based ETLs, a compact TiO_2_ (c-TiO_2_) layer was deposited via spray pyrolysis using a precursor solution composed of 800 μL of titanium diisopropoxide bis­(acetylacetonate) (75 wt % in IPA, Sigma-Aldrich) diluted in 10.8 mL of anhydrous ethanol (≥99.5%, Sigma-Aldrich). The solution was sprayed onto substrates preheated to 450 °C in cycles (16–18 s spray duration, 30 s pause between cycles), followed by annealing at 450 °C for 30 min. During spraying, O_2_ was used as the carrier gas at a flow rate of 3 L min^–1^. Once cooled to room temperature, a mesoporous TiO_2_ (mp-TiO_2_) layer was deposited by spin coating 60 μL of a 150 mg mL^–1^ TiO_2_ paste solution (diluted in anhydrous ethanol, Sigma-Aldrich) at 4000 rpm for 10 s (4000 rpm s^–1^). The mp-TiO_2_ layer was annealed at 100 °C for 10 min and then sintered at 450 °C for 30 min.

For NDI-based ETLs, NDI-(BnPA)_2_ and Br_2_-NDI-(BnPA)_2_ thin films were prepared by CBD. Cleaned, patterned FTO substrates were UV-ozone treated for 1 h before immersion in a preheated 0.5 mg mL^–1^ DMSO solution of the respective NDI-based molecule at 100 °C for 72 h. After deposition, substrates were dipped 3 times in ethanol and annealed at 120 °C for 10 min. All CBD steps were carried out in ambient air, after which the substrates were transferred to a N_2_-filled glovebox (O_2_ and H_2_O < 4 ppm).

The perovskite film, Cs_0.09_FA_0.91_PbI_3_ (CsFA), was deposited by using a two-step spin-coating method from a 1.2 M precursor solution with 5% excess Pb. The solution was prepared by dissolving cesium iodide (Sigma-Aldrich), formamidinium iodide (GreatCell Solar), and lead iodide (Tokyo Chemical Industry, >98%) in a 2:1 (v/v) mixture of DMF (Sigma-Aldrich, ≥99.8%) and DMSO. A 90 μL CsFA solution was spin-coated at 1000 rpm for 10 s (1000 rpm s^–1^), followed by 6000 rpm for 20 s (6000 rpm s^–1^). Three seconds before the end of the second step, 250 μL of CB (Sigma-Aldrich, 99%) was dynamically dispensed onto the substrate. The resulting CsFA film was annealed at 150 °C for 10 min. A passivation layer was applied by spin-coating 90 μL of phenethylammonium iodide (PEAI, Dyenamo) solution at 1 mg mL^–1^ in IPA (Sigma-Aldrich, anhydrous, 99.9%) at 5000 rpm for 20 s (5000 rpm s^–1^).

The HTL was deposited by spin coating 90 μL of doped 2,2′,7,7′-tetrakis­[*N*,*N*-di­(4-methoxyphenyl)­amino]-9,9′-spirobifluorene (spiro-OMeTAD) solution at 3000 rpm for 30 s (3000 rpm s^–1^). The spiro-OMeTAD solution was prepared by dissolving spiro-OMeTAD (1-Material) in 0.07 M CB (Sigma-Aldrich, 99.9%), followed by the sequential addition of lithium bis­(trifluoromethane)­sulfonimide (Li-TFSI, Sigma-Aldrich, 0.4 mol-to-mol) in 1.8 M acetonitrile, 4-*tert*-butylpyridine (tBP, Sigma-Aldrich, 98%, 3.3 mol-to-mol), and tris­(2-(1H-pyrazol-1-yl)-4-*tert*-butylpyridine)­cobalt­(III) tri­[bis­(trifluoromethane)­sulfonimide] (FK 209 Co­(III), Sigma-Aldrich, 0.03 mol-to-mol) in 0.25 M acetonitrile. All steps from perovskite deposition to HTL processing were carried out inside a N_2_-filled glovebox (O_2_ and H_2_O < 4 ppm) at a controlled temperature of 18–24 °C. The substrate edges were cleaned in ambient air using DMF followed by acetonitrile to remove the perovskite and HTL. Finally, a 50 nm Au layer (Kurt J. Lesker, 99.999%) was thermally evaporated through a shadow mask, defining eight individual cells per substrate with an active area of 0.128 cm^2^.

### ^1^H, ^13^C, and ^31^P NMR

NMR spectra for all monomers and molecular precursors were recorded using Bruker Avance III HD 500 or 700 MHz spectrometers. Deuterated chloroform (CDCl_3_) or deuterated DMSO (DMSO-d6) served as solvents, and chemical shifts were reference to the residual solvent signals (^1^H: δ= 7.26 ppm, ^13^C: δ= 77.16 ppm). For the ^31^P NMR spectra, 85% H_3_PO_4_ was used as the external reference.

### TGA

TGA was conducted on a Mettler Toledo TGA2 STAR System. Approximately 3 mg of each powdered molecule was heated from 50 to 800 °C at a temperature rate of 15 °C min^–1^ in a N_2_-rich atmosphere to monitor thermal stability and decomposition behavior.

### DSC

DSC was carried out using a TA Instruments DSC250 differential scanning calorimeter with heating and cooling rates of 10 °C min^–1^. Approximately 5 mg of each powdered molecule was sealed in standard aluminum pans and analyzed under a N_2_-controlled environment to prevent oxidative degradation and ensure thermal stability assessment. The temperature range for analysis was from 0 °C to either 250 or 275 °C, depending on the molecule.

### UV–Vis Spectroscopy

Absorption spectra were collected using a Cary 5000 UV–Vis NIR spectrophotometer. All measurements were performed in double-beam mode to eliminate background signal and correct for the optical contribution of the glass substrate, enabling precise analysis of ultrathin NDI-based molecular films. For solution UV–Vis, NDI derivatives were dissolved in DMSO at a concentration of 0.02 mg mL^–1^ and placed in quartz cuvettes with a 1 cm path length.

### XPS

XPS was performed using a Thermo Scientific K-Alpha system equipped with a monochromatic Al Kα X-ray source (hv = 1486.6 eV). The incident X-ray beam was directed at a 60° angle relative to the surface normal, while photoelectrons were collected along the surface normal (0° emission angle). All measurements were conducted under ultrahigh vacuum conditions with the chamber pressure maintained below 1 × 10^–7^ Torr. Both survey and high-resolution spectra were acquired. Survey scans were averaged over two measurements using a 200 eV pass energy, 50 ms dwell time, and 0.1 eV step size. High-resolution spectra were collected and averaged over 20 scans for the C 1s, Br 3d, P 2p, and O 1s regions; 10 scans for O 1s and N 1s; and 5 scans for Sn 3d. All spectra were processed and fitted using the Thermo Scientific Advantage Data System software. To compensate for surface charging, binding energies were calibrated using the C–C bond signal in the C 1s region set to 284.8 eV as the internal reference.

### UPS

UPS was performed using a Thermo Fisher Scientific Nexsa G2 system equipped with a HE I excitation source (21.22 eV). A −5 V sample bias was applied to clearly resolve the secondary electron cutoff (SECO), and spectra were acquired with a pass energy of 2 eV to ensure high resolution under ultrahigh vacuum conditions (<1 × 10^–8^ Torr). Prior to sample measurements, a Ag reference was used to calibrate the instrument by aligning its Fermi edge to 0 eV, establishing the binding energy offset for subsequent spectra. The SECO was determined by linear extrapolation of the low-kinetic-energy edge, and the work function was calculated as the difference between the photon energy and the SECO. The HOMO or VBM onset was extracted via linear fitting of the spectral onset near the Fermi level, and its energy relative to vacuum was determined by summing the binding energy onset with the measured work function. The intensity was converted to a logarithmic scale to more clearly identify the onset of the HOMO/VBM onset. UPS measurements were performed on thin films of NDI-(BnPA)_2_, Br_2_-NDI-(BnPA)_2_, and CsFA deposited on ITO substrates using deposition methods consistent with the mentioned PSC fabrication.

### Device Characterization

The photovoltaic performance of the devices was evaluated using a Fluxim Litos Lite measurement system coupled with a Wavelabs Sinus-70 AAA solar simulator, delivering AM1.5G spectral illumination at ambient temperature and atmosphere. *J–V* curves were acquired in both reverse and forward scanning directions over a voltage range from 1.2 V to −0.5 V, with a scan rate of 50 mVs^–1^. Stabilized PCE was determined via MPPT over a 120-s interval. A photomask was applied to define the illuminated areas as 0.0625 cm^2^, while the total active area was 0.128 cm^2^. Devices were measured under N_2_ flow without external temperature regulation, and no preconditioning, such as light soaking or biasing, was applied prior to testing.

For long-term stability studies, a Fluxim Litos stress-test system was used to track PCE degradation over time. Devices were continuously operated under MPPT conditions while exposed to 1 sun equivalent UV-filtered illumination in an inert N_2_ atmosphere at 25 °C, which adheres to the ISOS-L-1I protocol. *J–V* scans in both reverse and forward sweep directions were recorded automatically every 12 h to monitor changes in device parameters. For this stability measurement, a full device area of 0.128 cm^2^ was utilized.

## Supplementary Material



## Notes

Synthetic information and corresponding NMR data for all synthesized molecules, materials characterization data, and device performance metrics for perovskite solar cells (PDF)
