# Supplementary material for: Deepening the LUMO: Brominated Naphthalene Diimide Electron Transport Layers for Low-Hysteresis Perovskite Solar Cells
Source: Chem Mater. 2025 Nov 19;37(23):9479–86. doi: 10.1021/acs.chemmater.5c02132 (PMC12874372; doi:10.1021/acs.chemmater.5c02132)
Supplement: Supplementary file 1 [file cm5c02132_si_001.pdf]

Supplementary Information

Deepening the LUMO: Brominated Naphthalene Diimide Electron Transport Layers for Low-Hysteresis Perovskite Solar Cells

Sanggyun Kim[1], Justine S. Wagner[2], Sina Sabury[2], Spencer J. Gilman[2], Jack Lawton[1], D. Eric Shen[2], Anna M. Österholm[2], Carlo A. R. Perini[1], John R. Reynolds[1,2*], Juan-Pablo Correa-Baena[1,2*]

[1] School of Materials Science and Engineering, Georgia Institute of Technology, Atlanta, Georgia 30332, USA.

[2] School of Chemistry and Biochemistry, Center for Organic Photonics and Electronics, Georgia Institute of Technology, Atlanta, Georgia, 30332, USA.

Corresponding author: JPCB jpcorrea@gatech.edu; JRR jreynolds43@gatech.edu

ORCID:

Sanggyun Kim 0000-0002-0620-3090

Justine S. Wagner 0000-0003-4647-0449

Sina Sabury 0000-0003-0347-7405

Spencer J. Gilman 0000-0002-2837-2012

Jack Lawton 0009-0005-4672-1430

Dwanleen E. Shen 0000-0002-8318-898X

Anna M. Österholm 0000-0001-6621-8238

Carlo A.R. Perini 0000-0001-7582-2234

John R. Reynolds 0000-0002-7417-4869

Juan-Pablo Correa-Baena 0000-0002-3860-1149

Molecule Synthesis

Synthesis of tetraethyl (((1,3,6,8-tetraoxo-1,3,6,8-tetrahydrobenzo[lmn][3,8]phenanthroline-2,7-diyl)bis(4,1-phenylene))bis(methylene))bis(phosphonate), NDI-(BnDEP)$_2$:

1,4,5,8,-napthalenetetracarboxylic dianhydride (1.44 g, 5.36 mmol, 1 eq.) was added to a 250 mL round bottom flask followed by 80 mL of N,N-dimethylformamide. Then, (4-aminobenzyl)phosphonate (3.0 g, 12.3 mmol, 2.3 eq.) was added over the course of 10 minutes. The resulting mixture was heated to 120 °C and stirred overnight. Afterwards, the reaction was taken off the hot plate to cool down to room temperature. The solvent (N,N-dimethylformamide) was removed under high vacuum at 50°C. The obtained solid was purified using silica gel column chromatography with 89:10:1 ratio of dichloromethane: methanol: triethanolamine as mobile phase to afford pale yellow solid (3.74 g, 97%). ^{1}H NMR (500 MHz, DMSO) δ 8.72 (s, 4H), δ 7.43 (dd, J=12.8, 8.1 Hz, 8H), δ 4.03 (q, J=7.3 Hz, 8H), δ 2.81 (d, J =79.5 Hz, 4H), δ 1.24 (t, J =7.0 Hz, 12H) (**Fig. S3**). Carbon NMR could not be obtained due to solubility limit. ^{31}P NMR (500 MHz, DMSO) δ 26.91 (**Fig. S4**).

Synthesis of ((1,3,6,8-tetraoxo-1,3,6,8-tetrahydrobenzo[lmn][3,8]phenanthroline-2,7-diyl)bis(4,1-phenylene))bis(methylene))bis(phosphonic acid) NDI-(BnPA)$_2$:

NDI-(BnDEP)$_2$ (1.4 g, 1.95 mmol, 1 eq.) and 25 mL of anhydrous dichloromethane were added to a 100 mL round bottom flask equipped with a magnetic stir bar under an inert atmosphere. After stirring for 10 minutes at room temperature, bromotrimethylsilane (1.79 g, 11.7 mmol, 6 eq.) was added, and the reaction was left to stir overnight at room temperature. After 12 hours, 3 mL of methanol was added and the mixture was left to stir for an additional 3 hours. The solution was then concentrated to complete dryness and the powder was suspended in methanol and then

filtered. The product was then dried under high vacuum to receive an off-white powder (1.04 g, 88% yield). ^1H NMR (500 MHz, DMSO) δ 8.72 (s, 4H), δ 7.39 (dd, J=12.8, 8.1 Hz, 8H), δ 3.08 (d, J=21 Hz, 4H) (**Fig. S5**). Carbon NMR could not be obtained due to solubility limit. ^{31}P NMR (500 MHz, DMSO) δ 21.25 (**Fig. S6**).

2,6-Dibromonapthalene-1,4,5,8-tetracarboxylic dianhydride (DBNDA):

1,4,5,8,-napthalenetetracarboxylic dianhydride (5 g, 18.64 mmol, 1 eq.) was suspended in 100 mL of concentrated sulfuric acid in a 250 mL round bottom flask. Afterwards, 13.3 g of 1,3-dibromo-5,5-dimethylhydantoin (DBH) was added in small aliquots (~1 g) every 5 minutes and stoppered between each addition. The resulting suspension was heated to 80 °C and let to stir overnight. Afterwards, the reaction was taken off the hot plate to cool down to room temperature and then poured into crushed ice. The obtained yellow solid was isolated by filtration, washed with distilled water and methanol, and then dried under high vacuum. DBNDA was isolated and obtained as a pale-yellow solid (7.0 g, 88% yield). ^1H NMR (400 MHz, DMSO) δ 8.80 (s, 2H) (**Fig. S7**). ^{13}C NMR (101 MHz, DMSO) δ 158.45, 138.02, 134.97, 129.91, 127.90, 125.26, 124.76, 123.91, 40.60, 40.39, 40.18, 39.98, 39.77, 39.56, 39.35 (**Fig. S8**).

Tetraethyl (((4,9-dibromo-1,3,6,8-tetraoxo-1,3,6,8-tetrahydrobenzo[lmn][3,8]phenanthroline-2,7-diyl)bis(4,1-phenylene)bis(methylene)bis(phosphonate), Br_2-NDI-$(BnDEP)_2$:

DBNDA (2.0 g, 4.7 mmol, 1 eq.) and 50 mL of glacial acetic acid were added to a 150 mL round bottom flask. The reaction stirred at room temperature before diethyl (4-aminobenzyl)phosphonate (4.6 g, 18.8 mmoles, 4 eq.) was added. The reaction was then heated to 110 °C for about 10 minutes and then cooled to room temperature; note, it is important to adjust the time as needed to prevent

the solution from turning red, which signals that an undetermined side reaction has occurred, and the reaction must be discarded and restarted. The precipitate was filtered and washed with glacial acidic acid (1 x 20 mL) and distilled water (4 x 25 mL) and dried under high vacuum. The product obtained was a red powder (1.2 g, 30% yield). ^{1}H NMR (500 MHz, DMSO) δ 8.76 (s, 2H), 7.46 (dd, J=8.5, 2.4 Hz, 4H), 7.38 (d, J=8.1 Hz, 4H), 4.03 (dd, J=7.9, 6.8 Hz, 8H), 1.24 (t, J=7.1 Hz, 12H) (**Fig. S9**). ^{31}P NMR (500 MHz, DMSO) δ 26.93 **(Fig. S10**). Carbon NMR could not be obtained due to low resolution.

(((4,9-dibromo-1,3,6,8-tetraoxo-1,3,6,8-tetrahydrobenzo[lmn][3,8]phenanthroline-2,7-diyl)bis(4,1-phenylene))bis(methylene))bis(phosphonic acid), Br_2-NDI-(BnPA)$_2$:

Br_2-NDI-(BnDEP)$_2$ (1.1 g, 1.3 mmol, 1 eq.) and 25 mL of anhydrous dichloromethane were added to a 100 mL round bottom flask charged with a magnetic stir bar under an inert atmosphere. After stirring for 10 minutes at room temperature, bromotrimethylsilane (1.9 g, 12.7 mmol, 10 eq.) was added and the reaction was left to stir overnight at room temperature. After 12 hours, 3 mL of methanol was added and left to stir for an additional 3 hours. The solution was then concentrated to complete dryness and the powder was suspended in methanol and filtered. The product was then dried under high vacuum to receive a light lavender powder (0.85 g, 86% yield). ^{1}H NMR (500 MHz, DMSO) δ 8.77 (s, 2H), 7.44 (dd, J=8.4, 2.4 Hz, 2H), 7.35 (d, J=8.00 Hz, 2H), 3.32 (br, 4H) 3.15-3.08 (m, 4H) (**Fig. S11**). ^{31}P NMR (500 MHz, DMSO) δ 21.16 (**Fig. S12**) Carbon NMR could not be obtained due to low resolution.

Figures & Tables

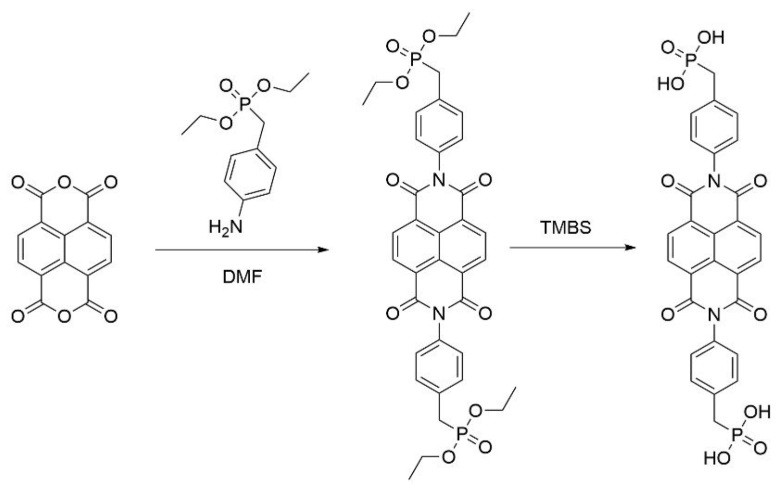

Figure S1. Synthetic scheme for NDI-$(BnPA)_2$.

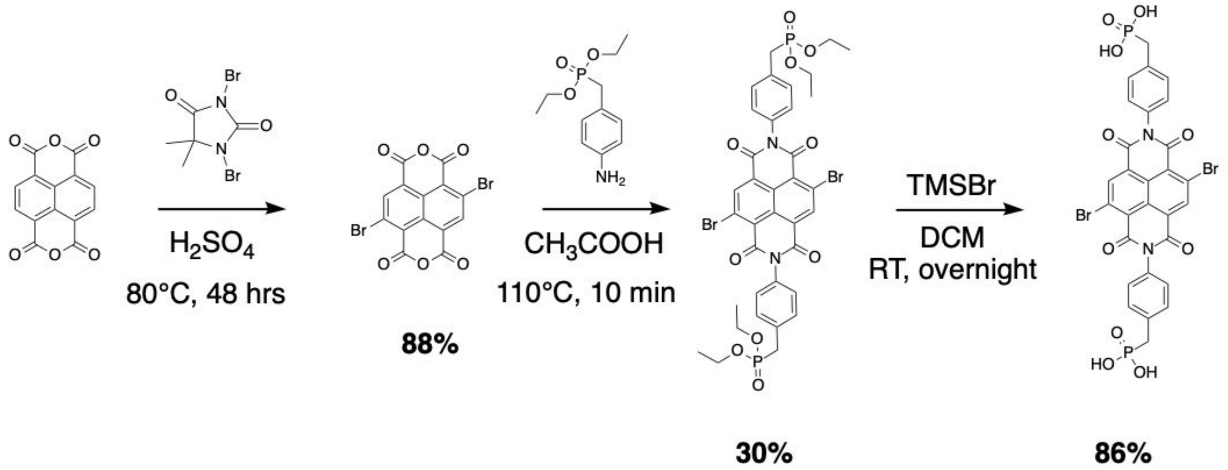

Figure S2. Synthetic scheme for Br_2-NDI-$(BnPA)_2$.

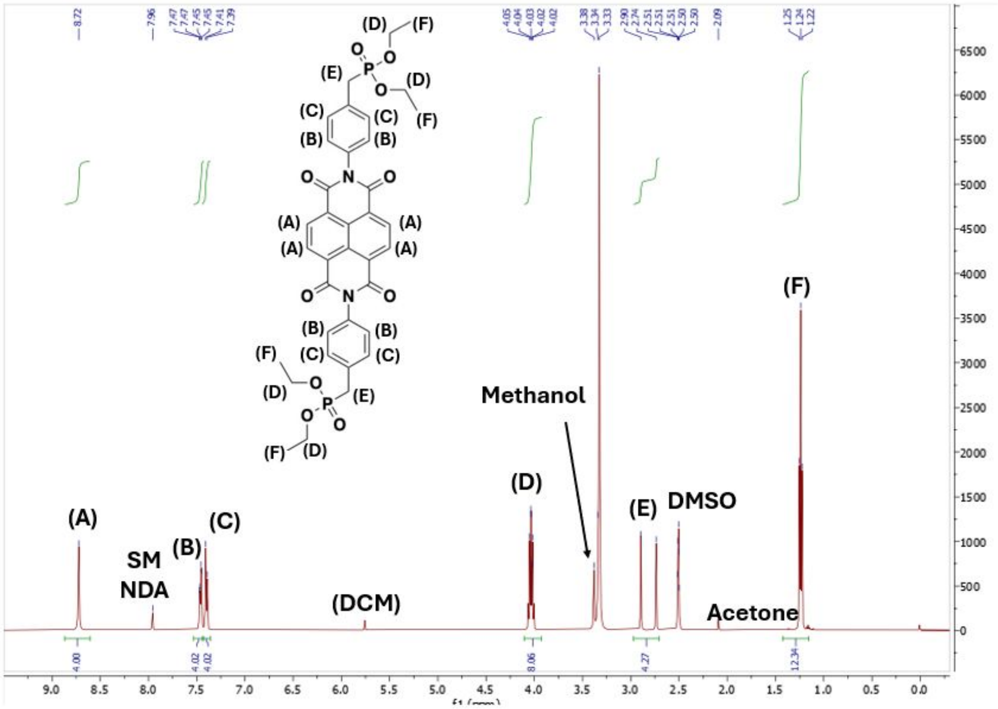

Figure S3. ^{1}H NMR (((1,3,6,8-tetraoxo-1,3,6,8-tetrahydrobenzo[lmn][3,8]phenanthroline-2,7-diyl)bis(4,1-phenylene))bis(methylene))bis(phosphonate), NDI-(BnDEP)$_2$.

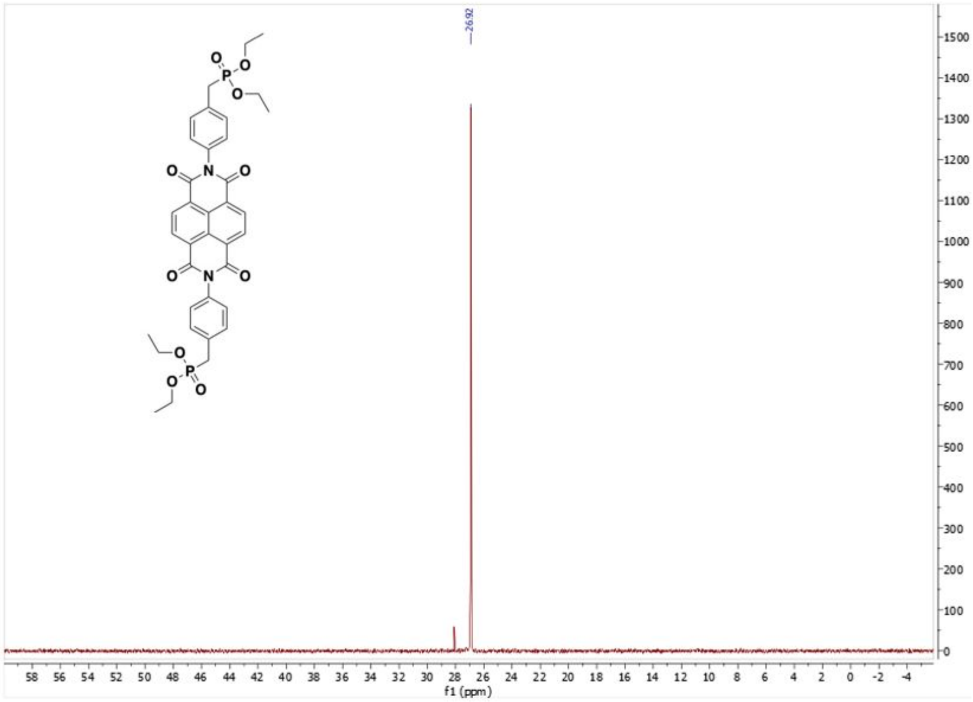

Figure S4. ^{31}P NMR (((1,3,6,8-tetraoxo-1,3,6,8-tetrahydrobenzo[lmn][3,8]phenanthroline-2,7-diyl)bis(4,1-phenylene))bis(methylene))bis(phosphonate), NDI-(BnDEP)$_2$.

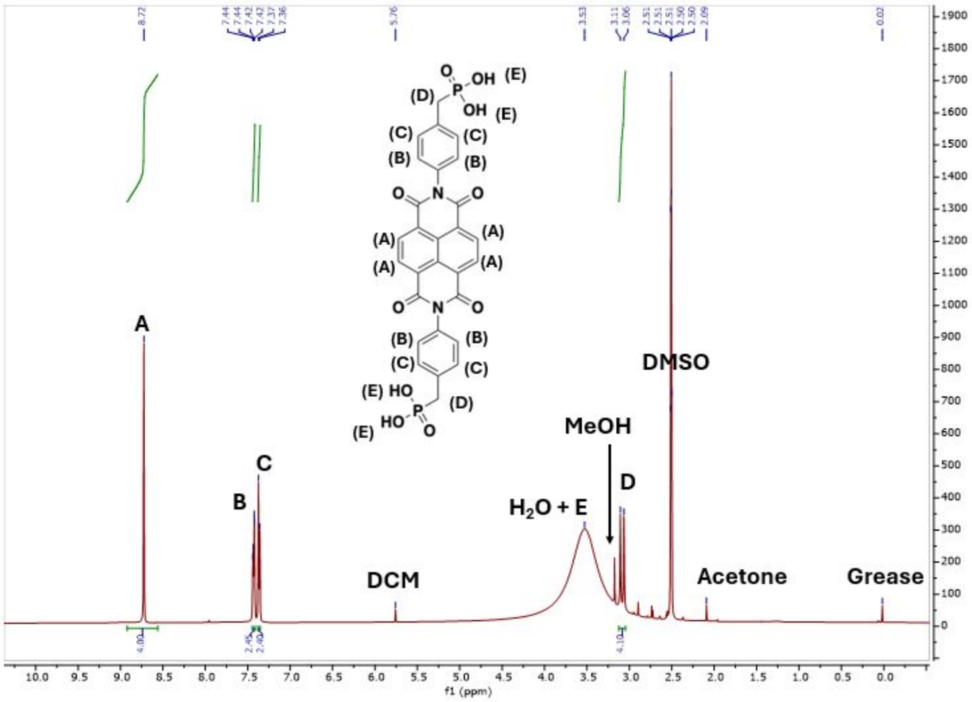

Figure S5. ^{1}H NMR ((1,3,6,8-tetraoxo-1,3,6,8-tetrahydrobenzo[lmn][3,8]phenanthroline-2,7-diyl)bis(4,1-phenylene))bis(methylene))bis(phosphonic acid), NDI-$(BnPA)_2$.

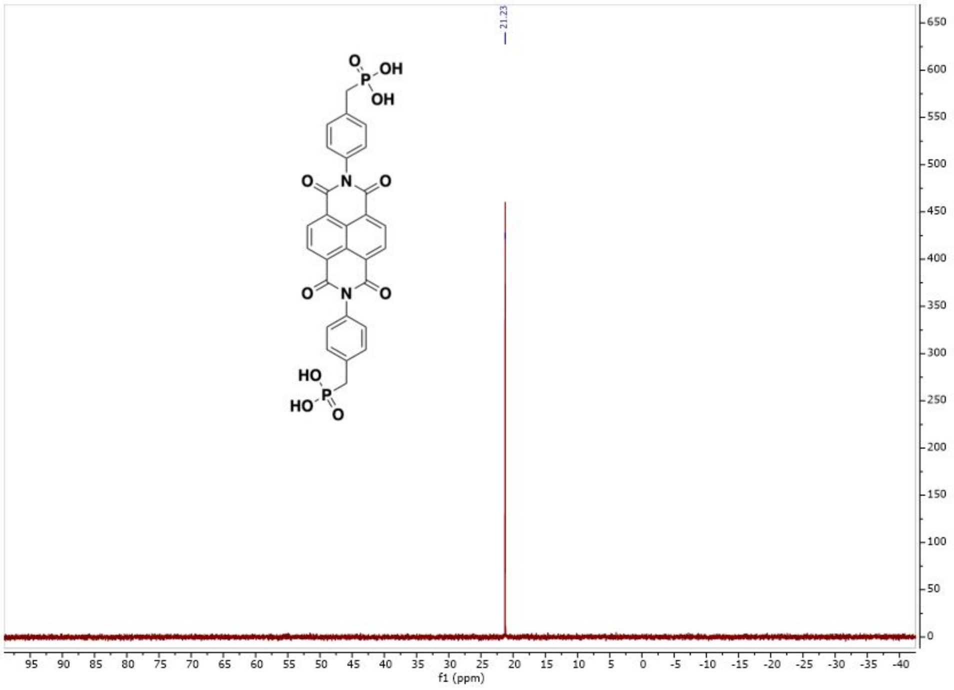

Figure S6. ^{31}P NMR ((1,3,6,8-tetraoxo-1,3,6,8-tetrahydrobenzo[lmn][3,8]phenanthroline-2,7-diyl)bis(4,1-phenylene))bis(methylene))bis(phosphonic acid), NDI-$(BnPA)_2$.

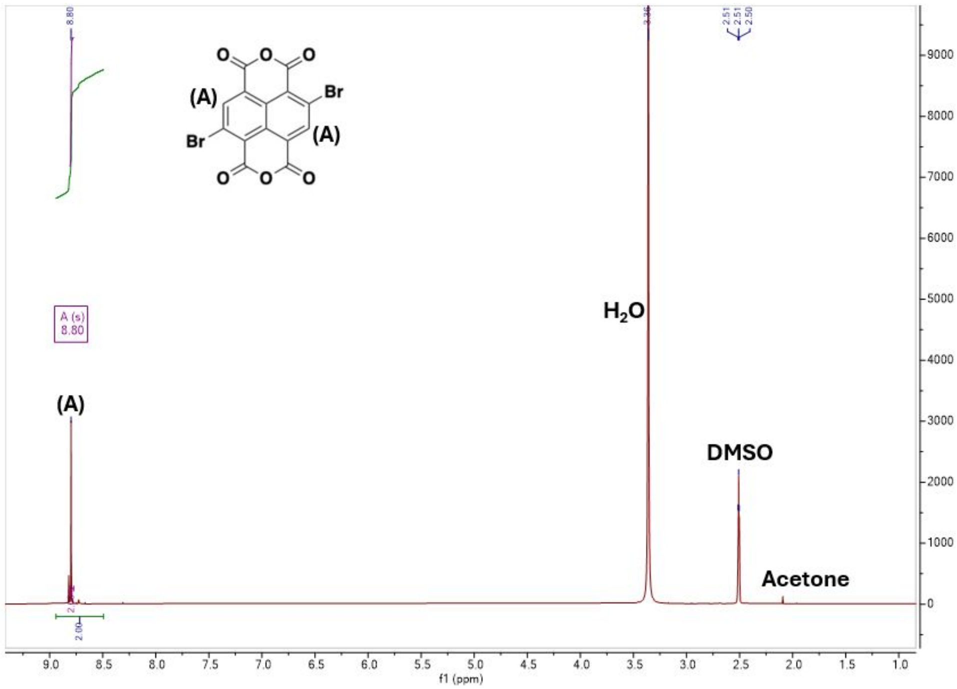

Figure S7. ^{1}H NMR 2,6-dibromonapthalene-1,4,5,8-tetracarboxylic dianhydride (DBNDA).

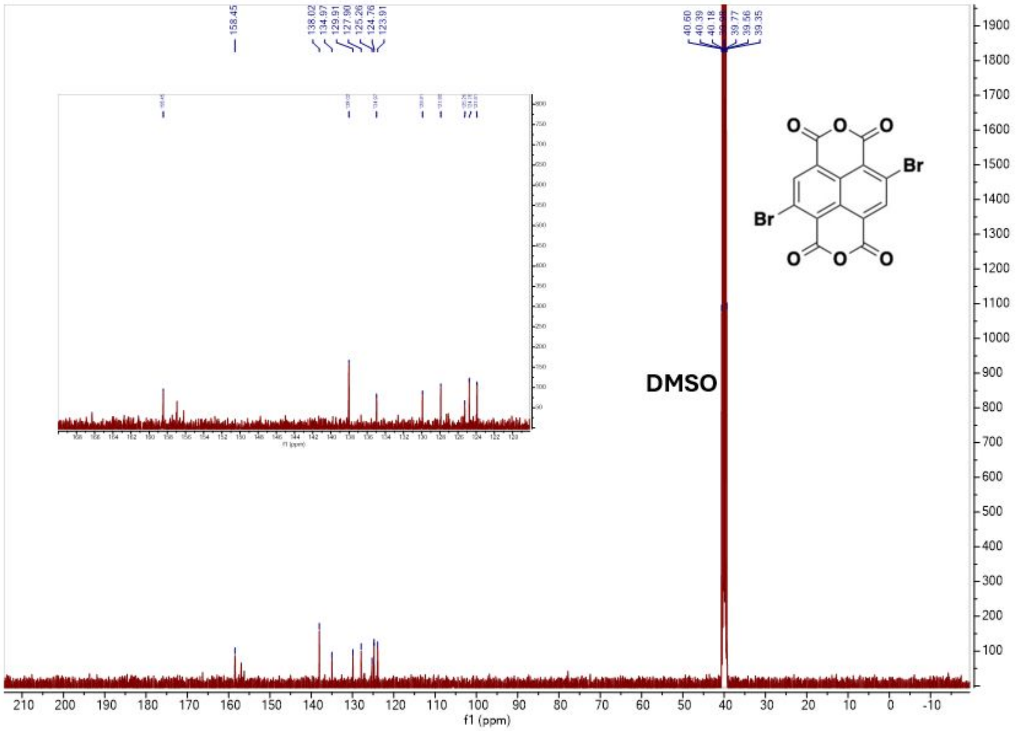

Figure S8. ^{13}C NMR 2,6-dibromonapthalene-1,4,5,8-tetracarboxylic dianhydride (DBNDA), including an inset with the enlarged 120-160 ppm range for clarity.

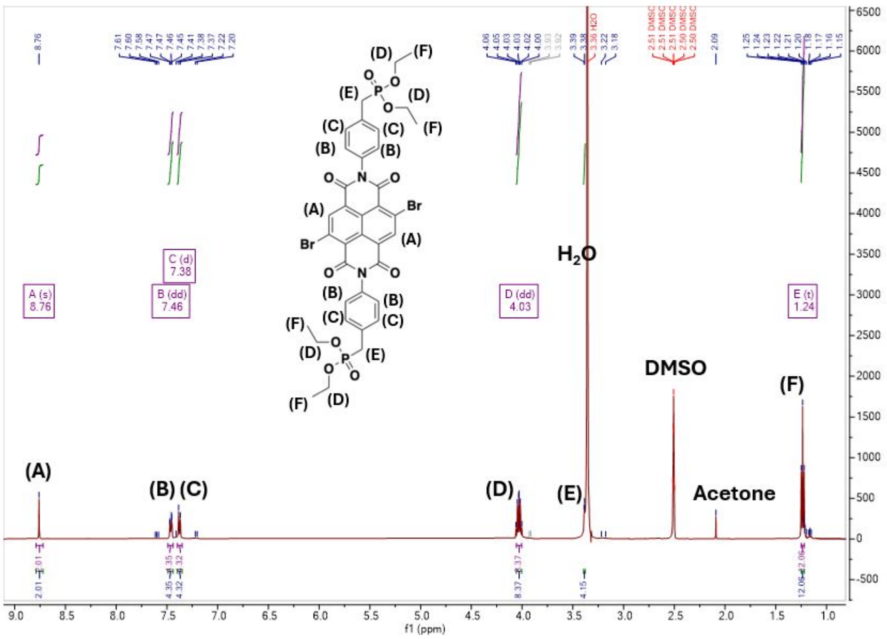

Figure S9. ^{1}H NMR of tetraethyl(((4,9-dibromo-1,3,6,8-tetraoxo-1,3,6,8tetrahydrobenzo [lmn][3,8] phenanthroline-2,7-diyl)bis(4,1-phenylene)bis(methylene)bis(phosphonate), Br_2-NDI-$(BnDEP)_2$.

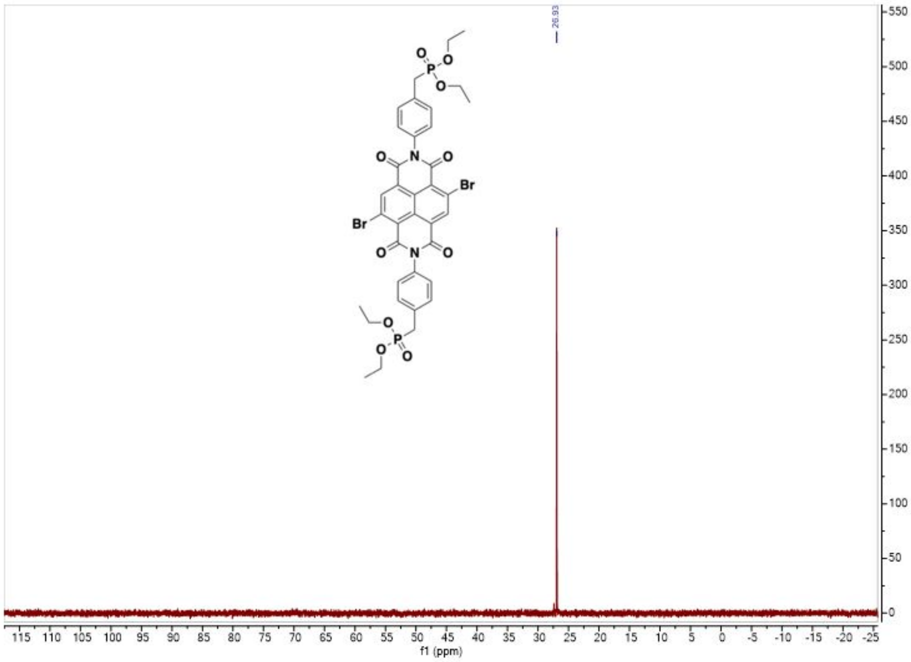

Figure S10. ^{31}P NMR of tetraethyl (((4,9-dibromo-1,3,6,8-tetraoxo-1,3,6,8-tetrahydrobenzo[lmn] [3,8]phenanthroline-2,7-diyl)bis(4,1-phenylene)bis(methylene)bis(phosphonate), Br_2-NDI-$(BnDEP)_2$.

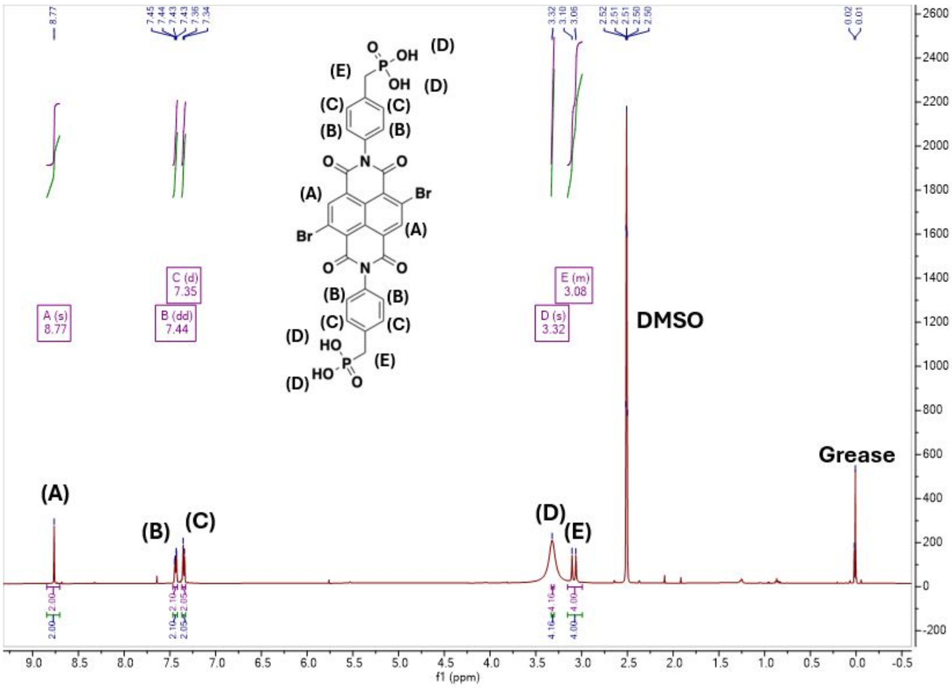

Figure S11. ^{1}H NMR of (((4,9-dibromo-1,3,6,8-tetraoxo-1,3,6,8-tetrahydrobenzo[lmn][3,8]phenanthroline-2,7-diyl)bis(4,1-phenylene))bis(methylene))bis (phosphonic acid), Br_2-NDI-$(BnPA)_2$.

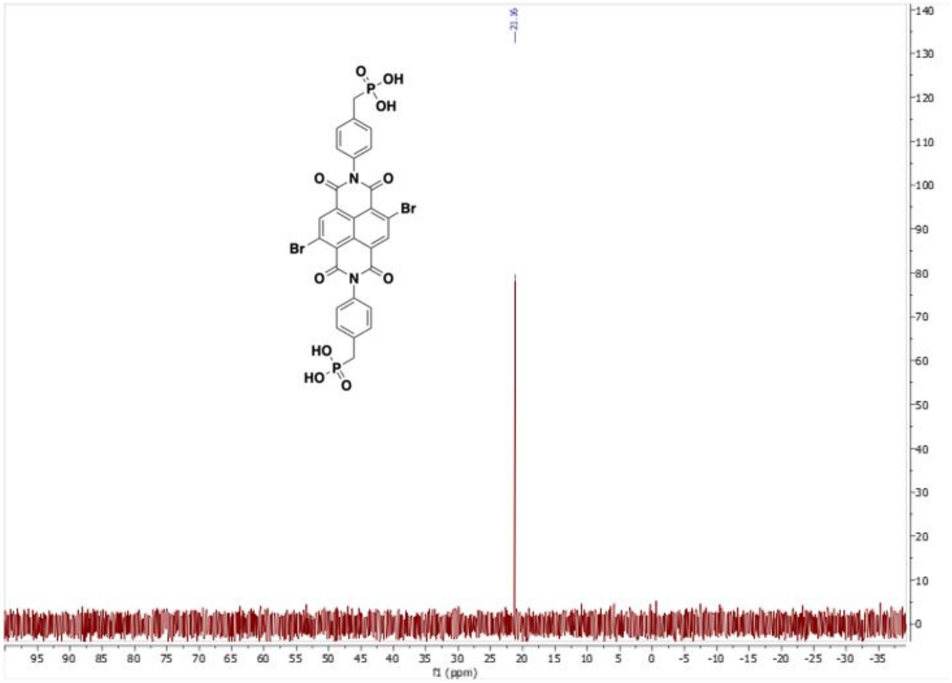

Figure S12. ^{31}P NMR of (((4,9-dibromo-1,3,6,8-tetraoxo-1,3,6,8-tetrahydrobenzo[lmn][3,8]phenanthroline-2,7-diyl)bis(4,1-phenylene))bis(methylene))bis (phosphonic acid), Br_2-NDI-$(BnPA)_2$.

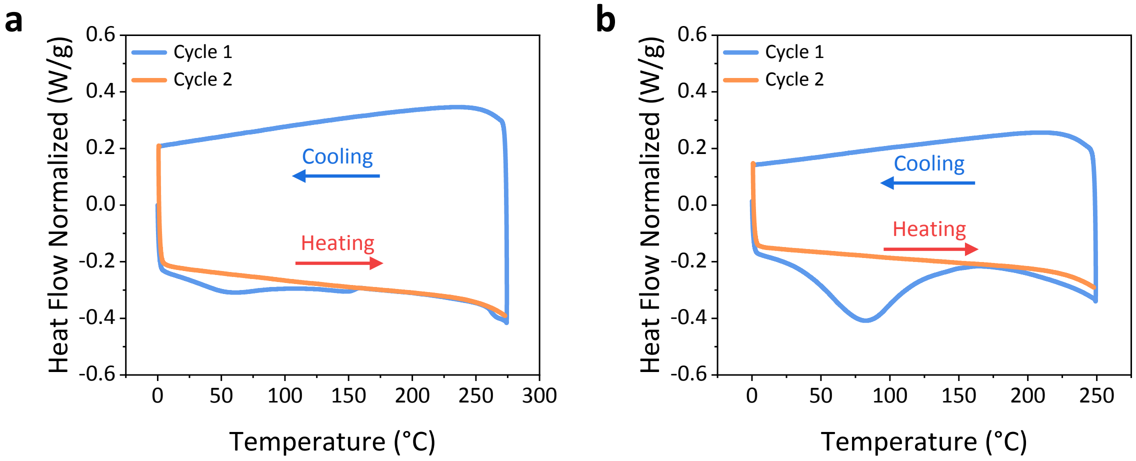

Figure S13. DSC thermograms of (a) NDI-$(BnPA)_2$ and (b) Br_2-NDI-$(BnPA)_2$ measured at scan rate of 10 °C min^{-1} in N_2 atmosphere. Heating and cooling cycles are shown to assess thermal transitions and potential phase behavior relevant to PSC processing and operation conditions.

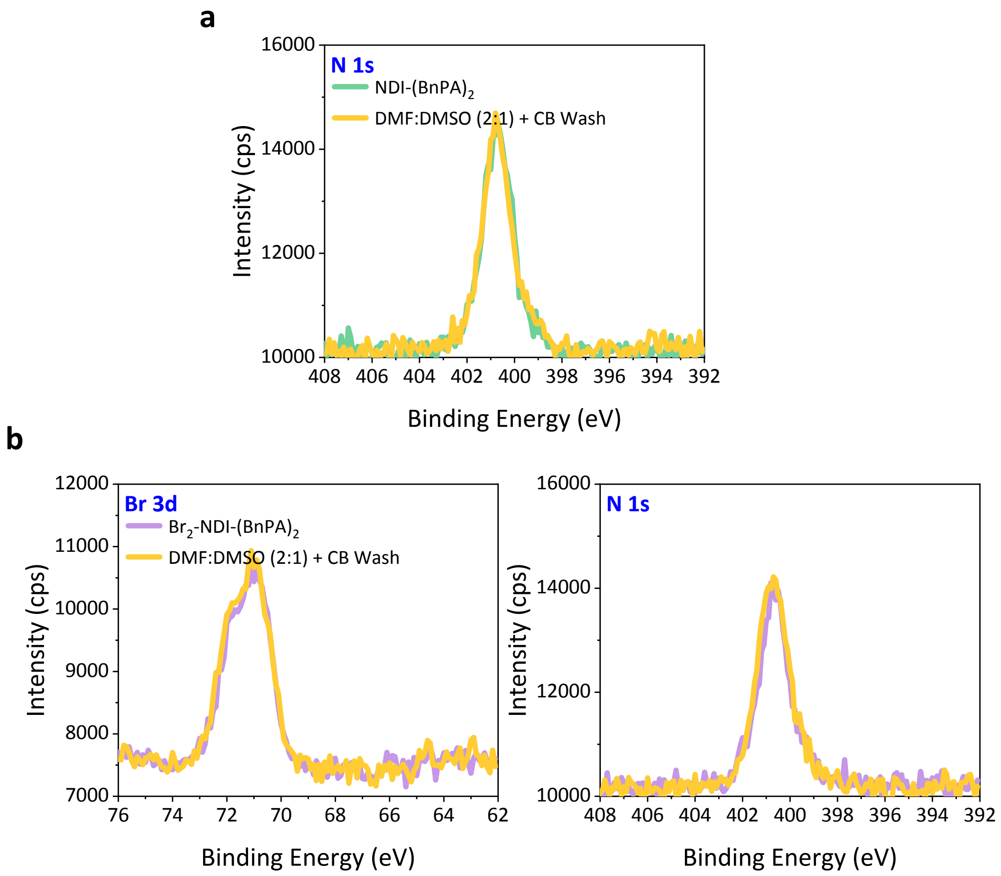

Figure S14. XPS spectra of (a) N 1s for NDI-$(BnPA)_2$, and (b) Br 3d and N 1s for Br_2-NDI-$(BnPA)_2$ on FTO, before and after washing with DMF:DMSO (2:1) + CB solvents, simulating the perovskite spin-coating conditions to assess molecular retention.

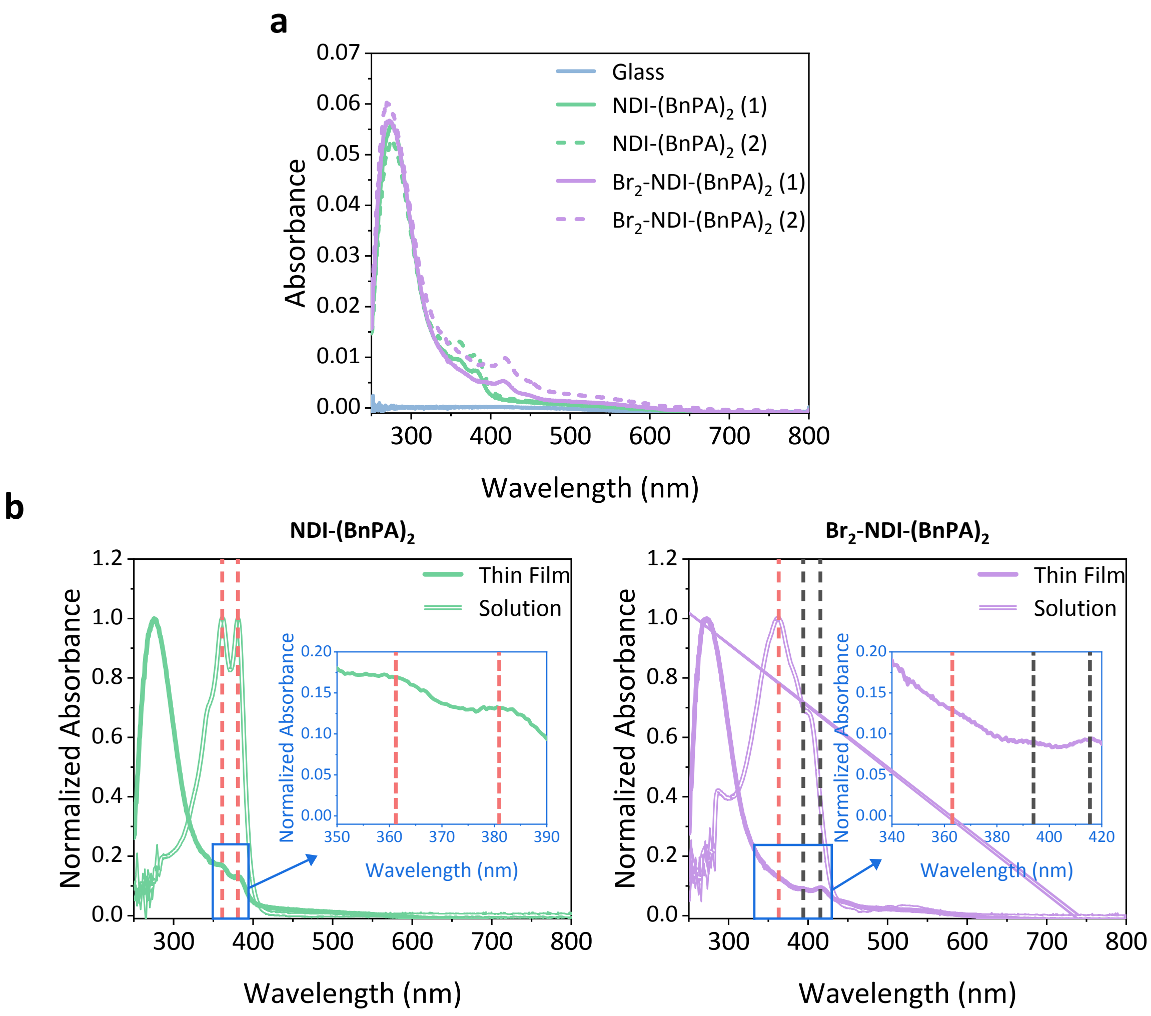

Figure S15. UV–Vis absorption spectra of NDI-$(BnPA)_2$ and Br_2-NDI-$(BnPA)_2$ in (a) non-normalized thin films and (b) normalized thin films and DMSO solutions. Red dashed lines denote absorption maxima in solution, which coincide with corresponding features in the thin films, indicating the preservation of molecular integrity during film formation. Insets provide magnified views of the spectral overlap. Black dashed line for Br_2-NDI-$(BnPA)_2$ shows the distinct absorption peaks from the thin films different from the solution UV-vis.

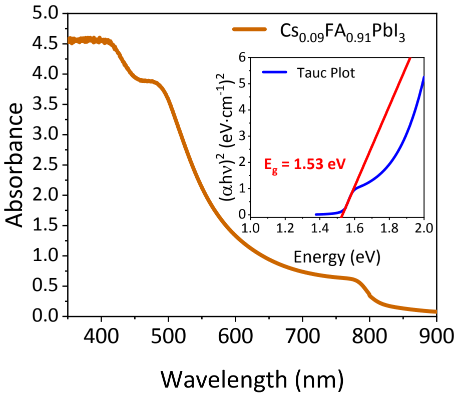

Figure S16. UV-Vis absorption spectra and optical energy band gap determination of $Cs_{0.09}FA_{0.91}PbI_3$ perovskite film glass substrate via Tauc method.

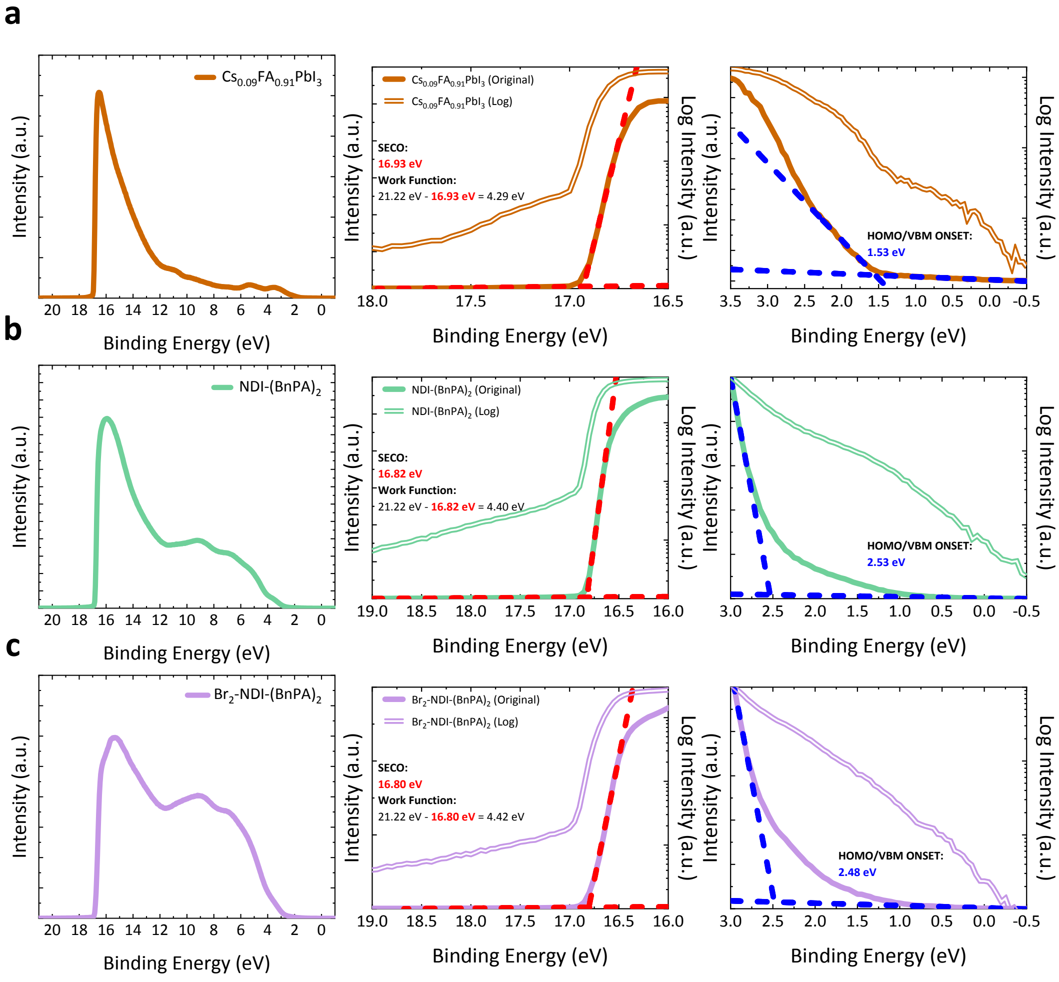

Figure S17. UPS spectrum, secondary electron cut-off (SECO), and highest-occupied molecular orbital (HOMO)/valence band maximum (VBM) onset of (a) $Cs_{0.09}FA_{0.91}PbI_3$ perovskite, (b) NDI-$(BnPA)_2$, and (c) Br_2-NDI-$(BnPA)_2$ on ITO. Work function and HOMO/VBM positions were determined from linear extrapolation SECO and onset edge, respectively.

Table S1. Optoelectronic properties of $Cs_{0.09}FA_{0.91}PbI_3$, NDI-$(BnPA)_2$, and Br_2-NDI-$(BnPA)_2$ on ITO determined by UV-Vis and UP measurements. Reported values for the work function, HOMO/VBM, and LUMO/CBM are referenced to the vacuum level.

Thin Film	$E_{HOMO-LUMO}$ or E_g (eV)	Work Function (eV)	HOMO/VBM (eV)	LUMO/CBM (eV)
$Cs_{0.09}FA_{0.91}PbI_3$	1.53	-4.29	-5.80	-4.29
NDI-$(BnPA)_2$	3.12	-4.40	-6.93	-3.81
Br_2-NDI-$(BnPA)_2$	2.80	-4.42	-6.90	-4.10

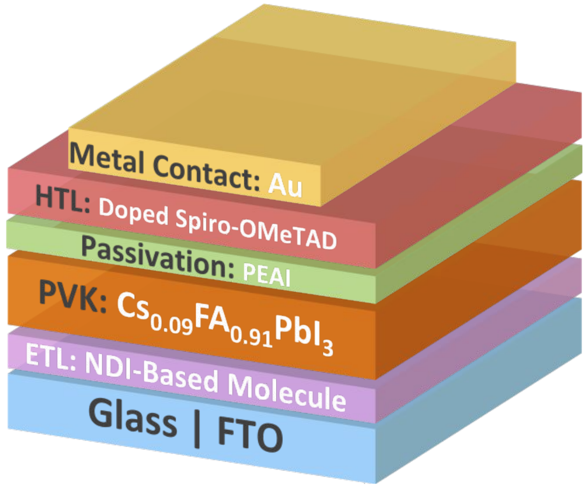

Figure S18. Device configuration of n-i-p PSC used in this work.

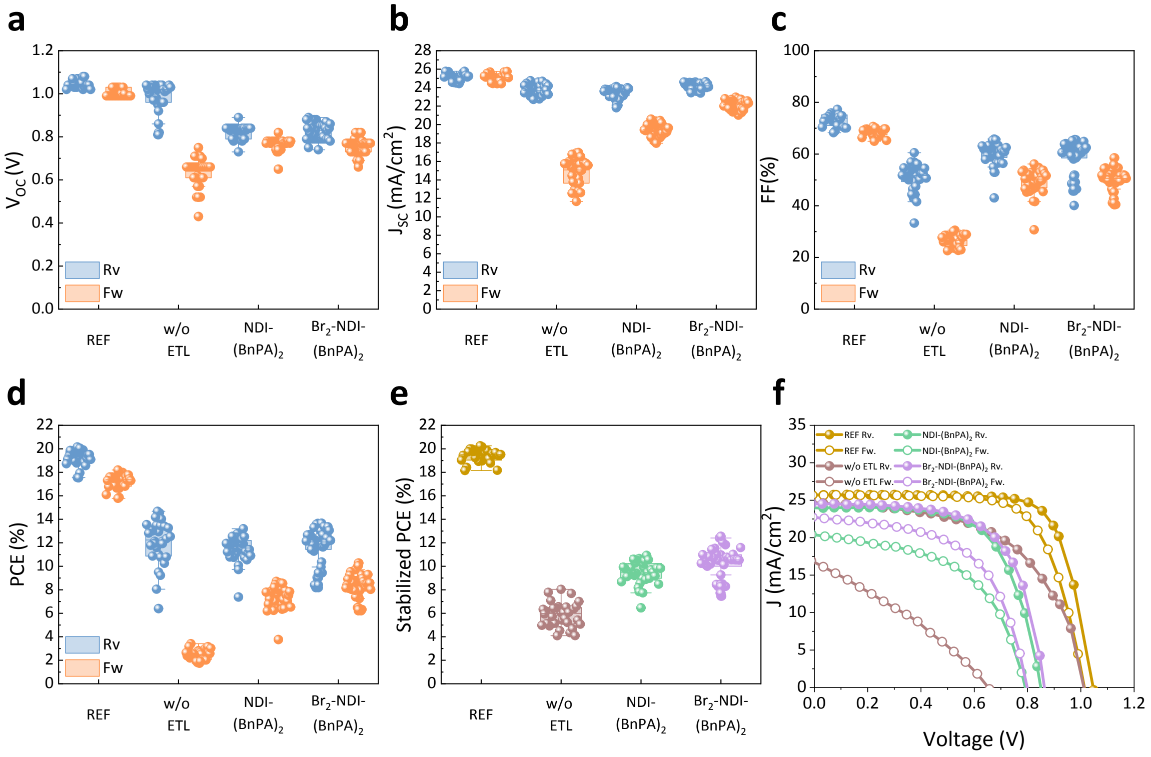

Figure S19. Statistics of (a) V_{OC}, (b) J_{SC}, (c) *FF*, (d) PCE, (e) stabilized PCE, and (f) *J-V* curves of champion devices for c-TiO_2 + mp-TiO_2 (REF), ETL-free (w/o ETL), NDI-(BnPA)$_2$, and Br_2-NDI-(BnPA)$_2$ incorporated PSCs.

Table S2. Summarized photovoltaic parameters for PSCs incorporating REF, w/o ETL, NDI-(BnPA)$_2$, and Br_2-NDI-(BnPA)$_2$. Data represents the average values and standard deviations from 24 cells for each variation.

ETL	Scan	V_{OC} (V)	J_{SC} (mA cm^{-2})	*FF* (%)	PCE (%)	Stabilized PCE (%)
REF	Rev.	1.04 ± 0.02	25.11 ± 0.38	73.89 ± 2.38	19.01 ± 1.28	19.39 ± 0.56
	Fwd.	0.99 ± 0.79	25.08 ± 0.38	68.46 ± 1.53	17.04 ± 1.00	
w/o ETL	Rev.	0.97 ± 0.13	23.68 ± 0.58	50.23 ± 6.40	11.78 ± 2.48	5.88 ± 1.03
	Fwd.	0.64 ± 0.07	14.80 ± 1.43	26.35 ± 2.14	2.48 ± 0.43	
NDI-(BnPA)$_2$	Rev.	0.82 ± 0.04	23.42 ± 0.54	59.87 ± 4.22	11.36 ± 1.35	9.44 ± 0.96
	Fwd.	0.73 ± 0.12	19.42 ± 0.67	49.25 ± 4.76	7.26 ± 1.26	
Br_2-NDI-(BnPA)$_2$	Rev.	0.81 ± 0.09	24.15 ± 0.34	58.98 ± 6.40	11.53 ± 2.08	10.29 ± 1.27
	Fwd.	0.74 ± 0.09	22.12 ± 0.46	50.20 ± 3.93	8.24 ± 1.45	

Table S3. Summarized hysteresis index for REF, w/o ETL, NDI-$(BnPA)_2$, and Br_2-NDI-$(BnPA)_2$.

ETL	Min. Hysteresis	Median Hysteresis
REF	0.08	0.10
w/o ETL	0.66	0.79
NDI-$(BnPA)_2$	0.26	0.36
Br_2-NDI-$(BnPA)_2$	0.13	0.29

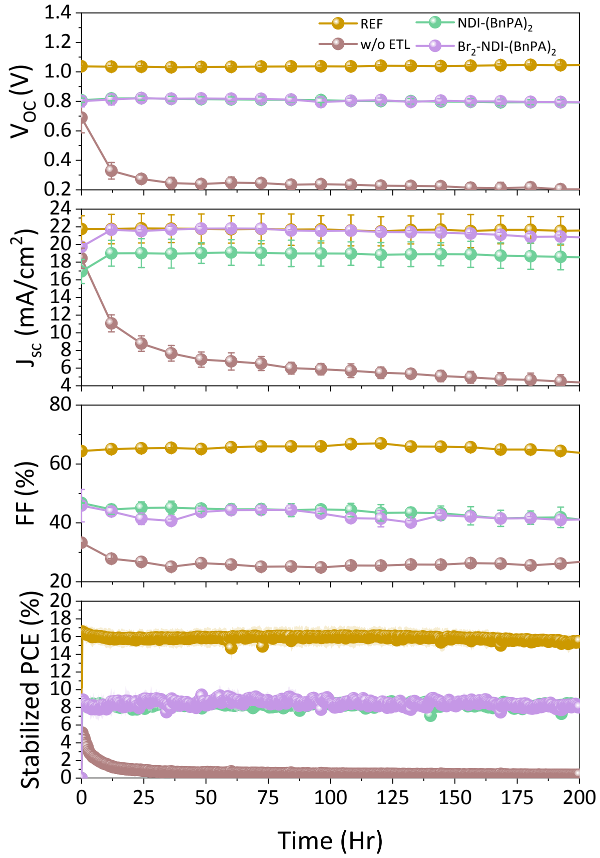

Figure S20. Evolution of the *J-V* parameters during long-term stability measurement of c-TiO_2 + mp-TiO_2 (REF), ETL-free (w/o ETL), NDI-$(BnPA)_2$, and Br_2-NDI-$(BnPA)_2$ incorporated PSCs. Photovoltaic parameters from *J-V* scans were automatically extracted every 12 hours during the MPPT at 25 °C.
